# The Abdominal Brain

**Published:** 1885-09

**Authors:** 


### BOOK NOTICES.

THE ABDOMINAL BRAIN. By Leila G. Bedell, M. D., Chicago. Gross & Delbridge, 1885.

This pamphlet of forty-five pages, as its title denotes, is devoted to an effort to elevate the importance of that portion of the nervous system distributed through the abdomen.

The authoress divides the nervous system of man into two grand divisions—the cerebro-spinal and the sympathetic systems: the former the system of animal life—"the outward, moving, thinking, seeing, feeling, hearing man"—dominated by the will and reason; the latter the system of organic life—performing the "functions of blood-making, blood-purifying, and blood-distributing"—dominated by the emotions.

The cerebro-spinal or animal system is concentrated in the brain, the reservoir of thought; the sympathetic or organic system is concentrated in the solar plexus, which is held to be the reservoir of the emotions. Hence the whole phenomena of emotion, love, hate, hope, fear, etc., arise only in the sympathetic system, and therefore within the abdomen. This is rather a startling theory, it must be said. The writer seeks to justify it, however, by quoting certain ancient popular beliefs, as that love exists in the heart, depression of mind and anger in the liver, spite and revenge in the spleen, compassion in the bowels.

Brain is that mass of nerve tissue, both gray and white, contained within the cranial cavity. It consists of the cerebrum, cerebellum, pons varolii, and medulla oblongata, with its prolongation, the spinal cord.

The solar plexus is the collection of ganglia or network of nerve filaments in the abdomen. The high importance of these plexuses in relation to life is an undisputed fact, but to call them the "Abdominal Brain" is an extravagant and fanciful expression, and to our mind absurd.

It is not possible to give a definition of mind, but mind originates in the brain proper. All the rest of the nerve system is subservient. Mind is the result of cerebral activity, largely influenced by the sympathetic system.

On page 6, these two systems are claimed to be "distinctly independent" and, therefore the whole man *dual* in his nature (p. 2). It is useless to call anything in the animal organism *dual.* The organism of necessity cannot be dual because all the functions are intimately associated with each other and are reciprocal. There cannot be any superiority of one set of functions over another in the organism, though one set may be more highly specialized than another. The cerebro-spinal and sympathetic systems are intimately related to each other, as the author says, but to set up two separate and independent systems, as the word *dualism* implies, is a certain error. The idea is objectionable because the two are inter-dependent. For example, the pneumogastric nerve is a cerebral nerve. It is intimately associated with the sympathetic, and at the same time has direct relation with life centres at the base of the brain. Therefore we hold it to be a cardinal error to say that the sympathetic alone controls the organic functions. The pneumogastric nerve has been anatomically demonstrated to inosculate with the sympathetic in the region of the solar plexus, hence, we repeat, it is an error to say that the sympathetic alone controls the functions of organic life.

The authoress hopes to be able to show which is the greater, the sympathetic or the cerebro-spinal system. This attempt will not be successful because there cannot be any question of supremacy between these two over the functions of the organism.

Again, the authoress says that all emotion has its origin in the sympathetic. Emotion is the result of external impressions which are received primarily in the cerebrum. Without doubt we appreciate them largely through the sympathetic system by transmission and reaction. Hence, to say that the sympathetic system is the seat and origin of emotions is to commit a fundamental error. Take, for example, the emotion of fear. A person sees a rattlesnake. The primary impression is made upon the cerebrum through the optic nerve, and the resulting emotion of fear is thrilled to every part of the body through both the sympathetic and the cerebro-spinal nerve fibres.

There is a meagre review of the comparative anatomy of the subject. On page 22 the writer endeavors to show the "antecedence" of the sympathetic system in race development. Among several forms noted she refers (p. 30) to *Amphioxus Lanceolatus.* If we consult Huxley's *Anatomy of Vertebrated Animals*, page 71, it will be found that "the sympathetic nervous system has *not* as yet been observed in the *Amphioxus.*"

At page 32 the writer says: "Point me out a single cranial or spinal nerve having sensory or centripetal fibres which has not upon its root at its very origin a ganglion of the sympathetic system." In answer to this we can state that three nerves of not only sensation but *special* sensation, the optic, olfactory, and auditory—sight, smell, and hearing—have no ganglia of the sympathetic in immediate connection with them. (*Vide* Huxley.)

In conclusion we may say that this pamphlet will serve a useful purpose in leading to thought upon the subject of the sympathetic system which may result in more extended investigations and throw more light upon the subject. W. M. J.

